# The altered intrinsic functional connectivity after acupuncture at shenmen (HT7) in acute sleep deprivation

**DOI:** 10.3389/fneur.2022.947379

**Published:** 2022-07-26

**Authors:** Yanzhe Ning, Sisi Zheng, Sitong Feng, Hao Yao, Zhengtian Feng, Xinzi Liu, Linrui Dong, Hongxiao Jia

**Affiliations:** ^1^The National Clinical Research Center for Mental Disorders and Beijing Key Laboratory of Mental Disorders, Beijing Anding Hospital, Capital Medical University, Beijing, China; ^2^Advanced Innovation Center for Human Brain Protection, Capital Medical University, Beijing, China

**Keywords:** large scale brain networks, sleep deprivation (SD), fMRI, functional connectivity, acupuncture

## Abstract

**Introduction:**

Accumulating evidence has shown that acupuncture could significantly improve the sleep quality and cognitive function of individuals suffering from insufficient sleep. Numerous animal studies have confirmed the effects and mechanisms of acupuncture on acute sleep deprivation (SD). However, the role of acupuncture on individuals after acute SD remains unclear.

**Methods:**

In the current study, we recruited 30 healthy subjects with regular sleep. All subjects received resting-state fMRI scans during the rested wakefulness (RW) state and after 24 h of total SD. The scan after 24 h of total SD included two resting-state fMRI sessions before and after needling at Shenmen (HT7). Both edge-based and large-scale network FCs were calculated.

**Results:**

The edge-based results showed the suprathreshold edges with abnormal between-network FC involving all paired networks except somatosensory motor network (SMN)-SCN between the SD and RW state, while both decreased and increased between-network FC of edges involving all paired networks except frontoparietal network (FPN)-subcortical network (SCN) between before and after acupuncture at HT7. Compared with the RW state, the large-scale brain network results showed decreased between-network FC in SMN-Default Mode Network (DMN), SMN-FPN, and SMN-ventral attention network (VAN), and increased between-network FC in Dorsal Attention Network (DAN)-VAN, DAN-SMN between the RW state and after 24 h of total SD. After acupuncture at HT7, the large-scale brain network results showed decreased between-network FC in DAN-VAN and increased between-network FC in SMN-VAN.

**Conclusion:**

Acupuncture could widely modulate extensive brain networks and reverse the specific between-network FC. The altered FC after acupuncture at HT7 may provide new evidence to interpret neuroimaging mechanisms of the acupuncture effect on acute SD.

## Introduction

With the rapid economic development, many individuals are undergoing insufficient sleep due to work or neuropsychological problems (1, 2). It has been reported that 30% of adults on average sleep <7 h per day (3). Sleep deprivation (SD) becomes a prevailing problem for many individuals in modern societies, leading to lower working performance, accidents in life, and a high risk of illness (4). In addition, SD interferes with the cognitive function and emotion of individuals (5). Due to acute SD, brain functions are damaged and subsequently promote the development of psychiatric and neurodegenerative diseases (6). It is critical that developing an efficacious intervention combats these negative consequences of SD.

There are several interventions for treating SD, such as pharmacological therapy, cognitive behavior therapy, and other complementary and alternative therapies (7). Acupuncture acts as the most important clinical treatment modality of traditional Chinese medicine, progressed through thousands of years in clinical practice (8). Accumulating evidence has shown that acupuncture could significantly improve the sleep quality and cognitive function of individuals suffering from insufficient sleep (9). Numerous animal studies had also confirmed the effects and mechanisms of acupuncture on acute SD (10, 11). Nonetheless, the role of acupuncture on individuals after SD remains unclear.

To test the role of acupuncture on individuals after acute SD, we first explored the single acupoint immediate effect on acute SD individuals. HT7 (Shenmen) is one of the most frequently used acupoints in improving sleep quality and cognitive impairments. Also, recent clinical studies have revealed that acupuncture at HT7 had a beneficial effect on sleep disorders among Chinese people (12). Furthermore, one animal study on rats after 72 h of SD revealed significant improvements in cognitive abilities and brainwaves after acupuncture at HT7 (10). However, little is known about the effect of acupuncture at HT7 on acute SD individuals.

The use of functional magnetic resonance imaging (fMRI) is effective to explore the effect of acupoint HT7 intervening acute SD. Increasing studies have investigated the aberrant activity of brain regions in diverse paradigms and imaging, concerning acute or chronic SD (13, 14). Specifically, brain-imaging studies have revealed that SD could convert the activities of several brain regions and change the connectivity of brain regions (15). Functional brain networks can display correlated activities when an individual is awake or at rest (16). Functional connectivity (FC) is the direct approach to calculate the connectivity between seed areas or brain networks (1). The altered FC within and between brain networks have been confirmed after SD, involving the Default Mode Network (DMN), Salience Network (SN), Dorsal Attention Network (DAN), and Frontoparietal Network (FPN) (17, 18). DMN is preferentially activated when people are engaged in internally-oriented tasks, e.g., daydreaming and retrieving memories (19). Numerous fMRI studies have revealed that SD reduces intrinsic connectivity within DMN and anti-correlated networks (i.e., Attention Network) (17, 20). After SD, the connection between FPN and DMN showed a decreased disposition (15, 18). SN is capable to detect and integrate emotional and sensory stimuli, regulating the DMN and central executive network (21). In short, numerous fMRI studies have reported the abnormal FC among these aforementioned large-scale functional brain networks in SD. However, to date, there was no study reporting FC changes between large-scale networks after acupuncture at HT7 on acute SD.

According to the controversial acupuncture modulation hypothesis, the acute SD subjects should be different from the RW subjects in intrinsic network FC, part of which would be reversed after acupuncture at HT7. In this study, we recruited 30 healthy subjects with regular sleep and detected 2 times resting-state fMRI scans during the rested wakefulness (RW) state and after 24 h of total SD. The scan after 24 h of total SD included two resting-state fMRI sessions before and after acupuncture at Shenmen (HT7). Both edge-based and large-scale network FCs were calculated between the RW state and after 24 h of total SD, before and after acupuncture at HT7, and RW state and after acupuncture at HT7.

## Materials and methods

### Subjects

Thirty healthy subjects (14 females) studying in the college, aged 20–30 years (25.20 ± 2.20 years) with an education duration of 18.10 ± 2.45 years, were recruited. All subjects must meet the following criteria: (1) had no symptoms associated with sleep disorders and Pittsburgh Sleep Quality Index score < 5; (2) normal sleep patterns and not extreme morning or evening types according to Horne-Ostberg Morningness-Eveningness Questionnaire; (3) right-handed; (4) no history of neurologic or psychiatric disorders; (5) no history of trauma stimuli for the latest 1 year; (6) no addiction of coffee, smoking, and alcohol; (7) no MRI contraindications. Our study protocol had been approved by the Ethics Committee of Beijing Anding Hospital. All enrolled subjects were required to sign the informed consent before the beginning of this study.

### Study procedure

**Figure 1:**
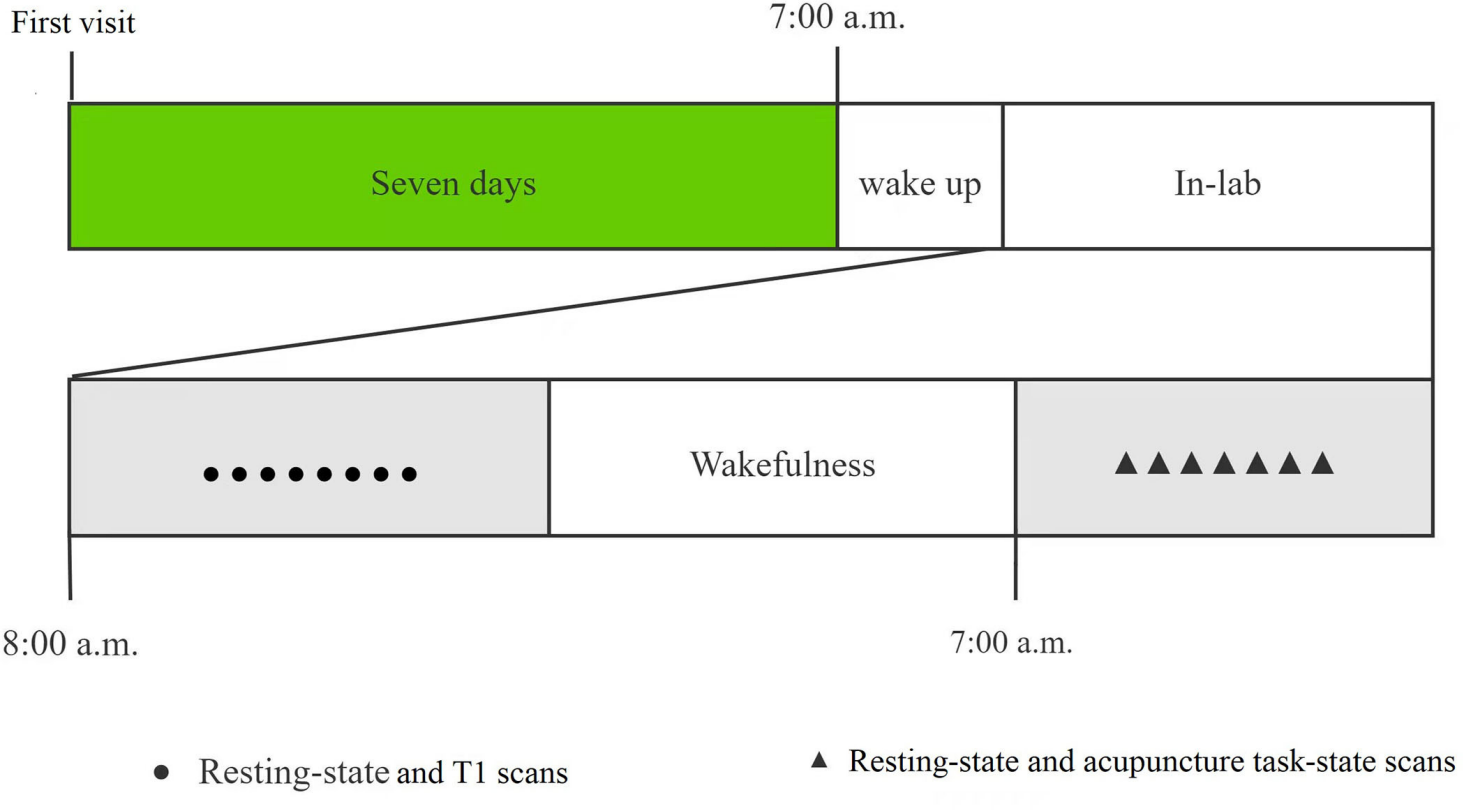
The flowchart of study procedure.

All recruited subjects were required to visit our laboratory twice. A brief introduction to the study protocol was provided and signed the informed consent at the first visit. During the second visit after a week, the subjects returned to the laboratory for 24 h SD from 8:00 am on the 1st day to 8:00 am on the 2nd day. During the SD, all subjects were required to stay awake all the time and not take tea, coffee, or alcohol. The members of our research group monitored in turns, to prevent subjects from falling asleep. They would be waked up immediately if they showed any signs of falling asleep. All subjects were required to complete twice MRI scans at the beginning of the study and after 24 h of SD. At the first MRI scanning, we performed the 490-s resting-state and 250-s T1 scans, while the 490-s resting-state and 550-s acupuncture task-state scan at the second MRI scanning after 7:00 am the next day. We would remind the participants not to fall asleep during scanning before each scan sequence, and rule out the participant who reported falling asleep during the fMRI scan. The study procedure was shown in Figure 1.

### Acupuncture task-state fMRI design

In our study, the non-repeated event-related fMRI design was employed to detect the effects of acupuncture, which was in line with our previous studies (22, 23). According to our previous study, we performed a 60-s acupuncture manipulation and then a 490-s resting-state scan (without manipulation). We would compare the two resting-state scans before and after acupuncture at HT7 to detect the acupuncture effect on SD.

Acupuncture was performed at bilateral Shenmen (HT7, located on the palmar ulnar end of the transverse crease of the wrist and on the radial aspect of the tendon of the ulnar flexor). The needling was conducted by inserting two sterile, single-use silver needles (0.5 mm in diameter and 40 mm in length) vertically into bilateral HT7s to a depth of 20–30 mm. The needling operation included rotating the needle clockwise and counterclockwise at 1 Hz with even reinforcing and reducing manipulation for 60 s. All needling operations were conducted by the same two licensed and skilled acupuncturists.

### MRI acquisition

In this study, we employed a 3.0 Tesla Prisma MRI scanner (Siemens) to acquire MRI scans at Beijing Anding Hospital. Subjects were required to stay still, keep their eyes closed, and refrain from falling asleep during the scan. Meanwhile, the foam head holders were immobilized to reduce head movements.

The high-resolution structural information for anatomical localization was obtained by applying 3D MRI sequences before the functional scanning. The resting-state and acupuncture task-state fMRI data were collected with a single-shot, gradient-recalled echo-planar imaging sequence with the following parameters: echotime = 30 ms, repetition time = 2,000 ms, flip angle = 90°, matrix = 64 × 64, gap = 1 mm, field of view = 225 × 225 mm, slice thickness = 3.5 mm, 32 interleaved axial slices and 180 volumes. The high-resolution structural scan was acquired with the following parameters: voxel size = 1 mm^3^, TR = 2,530 ms, TE = 3.39 ms, flip angle = 90°, matrix = 256 × 256, field of view = 256 × 256 mm, slice thickness = 1 mm.

### Data processing

All data processing was completed by DPABI (24) with the methods in the article published by Li et al. (25).

#### Anatomical data preprocessing

The T1 images were converted into the BIDS dataset. Then they were corrected for intensity non-uniformity with N4BiasFieldCorrection (26), which was provided by ANTs 2.3.3. The derived images were skull-stripped with OASIS30ANTs as the target template. The remaining brain tissues were segmented into the cerebrospinal fluid (CSF), white-matter (WM), and gray-matter (GM) by the BET (FSL 5.0.9). A classic method, which reconciles ANTs- and FreeSurfer-derived segmentation of the cortical gray-matter of Mindboggle (27), was applied for refining the brain mask estimated previously. Volume-based spatial normalization to one standard space (MNI152NLin2009cAsym) was conducted *via* non-linear registration with antsRegistration (ANTs 2.3.3), using brain-extracted versions of T1 reference and template. Meanwhile, we selected ICBM 152 Non-linear Asymmetrical template version 2009c for spatial normalization.

#### Functional data preprocessing

First, we used the custom methodology of fMRIPrep (28) to generate the reference volume and skull-stripped version. Susceptibility distortion correction (SDC) was omitted. Bbregister, which implements boundary-based registration, was applied for co-registering the fMRI reference and T1 reference. Moreover, slice-time was corrected using 3dT shift from AFNI and spatiotemporal filtering was conducted by mcflirt (FSL). The BOLD time series were resampled into standard space and generated a preprocessed BOLD run in MNI 152 NLin2009c Asym space. At the same time, framewise displacement (FD), DVARS, and three region-wise global signals were calculated by the preprocessed BOLD. Additionally, a set of physiological regressors were extracted to allow for component-based noise correction (CompCor). Above components were dropped from the BOLD and frames that exceeded a threshold of 0.5 mm FD or 1.5 standardized DVARS were annotated as motion outliers. Gridded (volumetric) resampling was performed using ants Apply Transforms (ANTs), configured with Lanczos interpolation to minimize the smoothing effects of other kernels. We used bandpass filter (0.01–0.08 Hz) to reduce the high-frequency physiological noise and low-frequency drift.

#### Edge-based FC calculation

The Dosenbach atlas (29), which contained 160 regions of interest (ROIs) and deleted 18 ROIs in the cerebellum, was selected to extract the BOLD signals which averaged across all voxels in the ROIs. Each node of the atlas was a 5 mm-radius sphere. Pearson's correlation coefficient of the BOLD signals was computed to define the FC for any pair of two ROIs. Meanwhile, the value of FC was transformed into z-scores by Fisher's r-to-z formula. Network-Based Statistic (NBS) with paired *T*-tests were applied to compare the FC inter and intra group (*p* < 0.01, two-tailed, permutation with 5,000 iterations). For exploring the relationship of each large-scale network, we classified suprathreshold edges by their membership in the networks according to Li et al. (25) and Yeo et al. (30).

#### Large-scale network FC calculation

The seven networks are the visual network (VN, 22 ROIs), subcortical network (SCN, seven ROIs), DAN (14 ROIs), FPN (21 ROIs), somatosensory-motor network (SMN, 29 ROIs), ventral attention network (VAN, 16 ROIs), and DMN (33 ROIs). Finally, we counted the number of edges within these networks and between these networks. Besides, we verified the above results by using a large-scale network FC analysis. Averaging the FC z-scores across all involved edges was applied for evaluating the FC among the seven networks. *T*-tests were used to compare the FC within and between groups (*p* < 0.05, False Discovery Rate Correction).

## Results

### Edge-based FC

**Figure 2:**
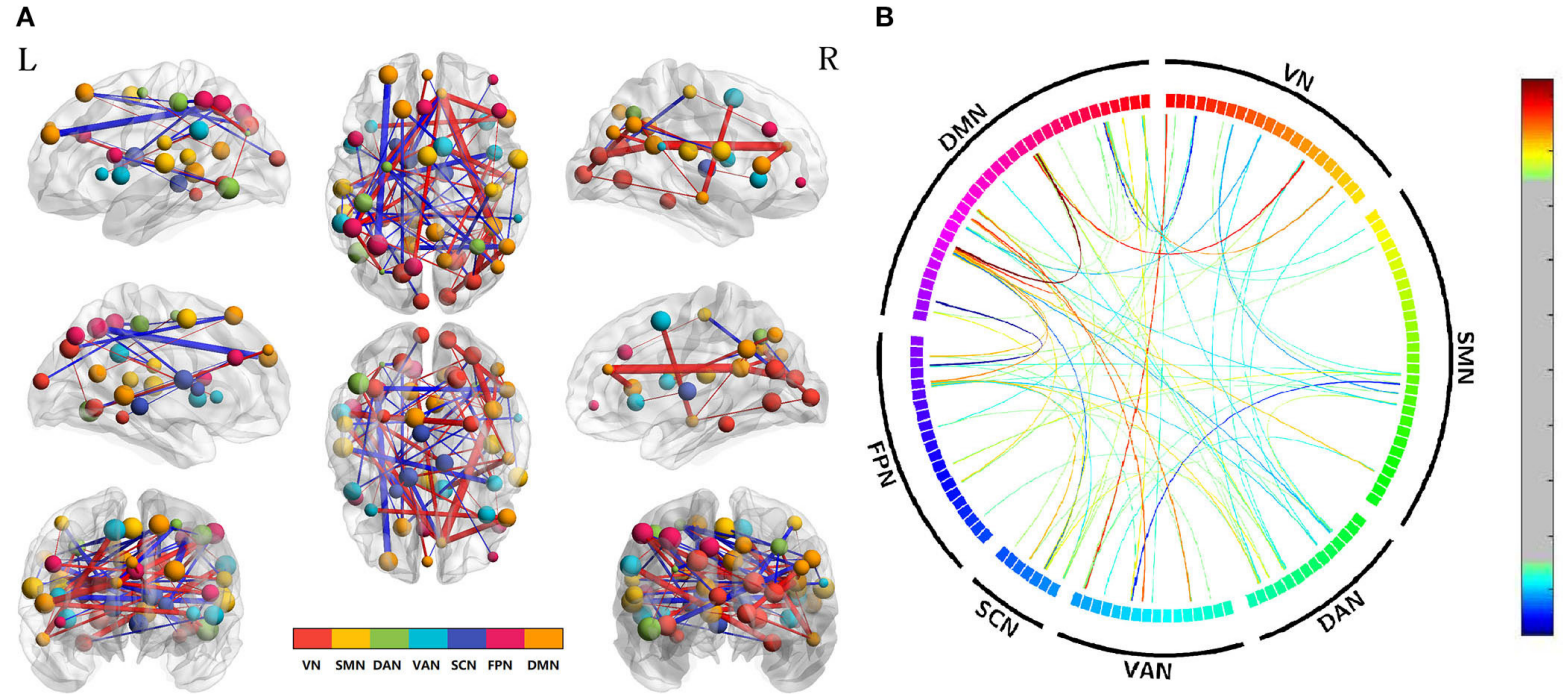
Altered edge-based functional connectivity between the acute SD and RW states. **(A)** The brain maps show the affected edges (lines) and their connecting nodes (spheres) from several perspectives. The size of a node indicates how many affected edges are connected to this sphere. Bigger nodes have more affected edges than smaller ones. The color of a node indicates which network it belongs to. The warm color of edges indicates the increased FC after acute SD, while the cold color of edges indicates the decreased FC. **(B)** ROI to ROI connectivity of seven networks in panels. The result was corrected by NBS (*p* < 0.01, two-tailed, permutation with 5,000 iterations). DAN, dorsal attention network; DMN, default mode network; FC, functional connectivity; FPN, frontoparietal network; L, left; R, right; SCN, subcortical network; SMN, somatosensory network; VAN, ventral attention network; VN, visual network; RW, rested wakefulness; SD, sleep deprivation.

NBS analysis (*p* < 0.01, two-tailed, permutation with 5,000 iterations) was used to calculate the edge-based FC among 142 ROIs. After conducting the differences in edge-based FC between the acute SD and RW states, we found that the suprathreshold edges with abnormal within-network FC involving DAN, SCN, DMN, and between-network FC involving all paired networks except SMN-SCN, which suggested the widespread impact of SD on brain networks. Most of the affected ROI connections between two networks included both decreased FC and increased FC of edges. While there was only decreased ROI-wise FC between networks involving VN-DAN and SMN-VAN. The only increased ROI-wise FC between networks included SMN-DAN, DAN-VAN, DAN-SCN, VAN-SCN, and VAN-DMN. Moreover, we found that SD subjects demonstrated only increased within-network FC of the SCN, while both increased and decreased within-network FC of the DAN and DMN. The details are shown in Table 1 and Figure 2.

**Table 1:** Number and ratio of decreased and increased ROI-wise FC between the acute SD and RW states.

**Number**	**VN**	**SMN**	**DAN**	**VAN**	**SCN**	**FPN**	**DMN**
**Percent**							
**Decreased ROI-wise FC**							
VN	0						
	0%						
SMN	2	0					
	0.3%	0%					
DAN	4	0	1				
	1.3%	0%	1.1%				
VAN	1	3	0	0			
	0.28%	0.65%	0%	0%			
SCN	0	0	0	0	0		
	0%	0%	0%	0%	0%		
FPN	3	3	2	1	1	0	
	0.65%	0.49%	0.68%	0.30%	0.68%	0%	
DMN	2	3	4	0	1	1	2
	0.28%	0.31%	0.87%	0%	0.14%	0.14%	0.38%
**Increased ROI-wise FC**							
VN	0						
	0%						
SMN	1	0					
	0.16%	0%					
DAN	0	1	1				
	0%	0.25%	1.1%				
VAN	1	0	1	0			
	0.28%	0%	0.45%	0%			
SCN	0	0	1	2	1		
	0%	0%	1.02%	1.79%	4.76%		
FPN	1	2	2	1	1	0	
	0.22%	0.33%	0.68%	0.3%	0.68%	0%	
DMN	3	1	3	3	4	3	6
	0.41%	0.10%	0.65%	0.57%	1.73%	0.43%	1.14%

*Values in the first line of each cell are the count number of suprathreshold edges belonging to each pair of networks for the significant cluster obtained from the NBS analysis; while values in the second line are the ratio (in percent) of that number to the number of full connections for each pair of networks. VN, visual network; SMN, somatosensory network; DAN, dorsal attention network; VAN, ventral attention network; SCN, subcortical network; FPN, frontoparietal network; DMN, default mode network, FC, functional connectivity; RW, rested wakefulness; SD, sleep deprivation; ROI, region of interest*.

**Figure 3:**
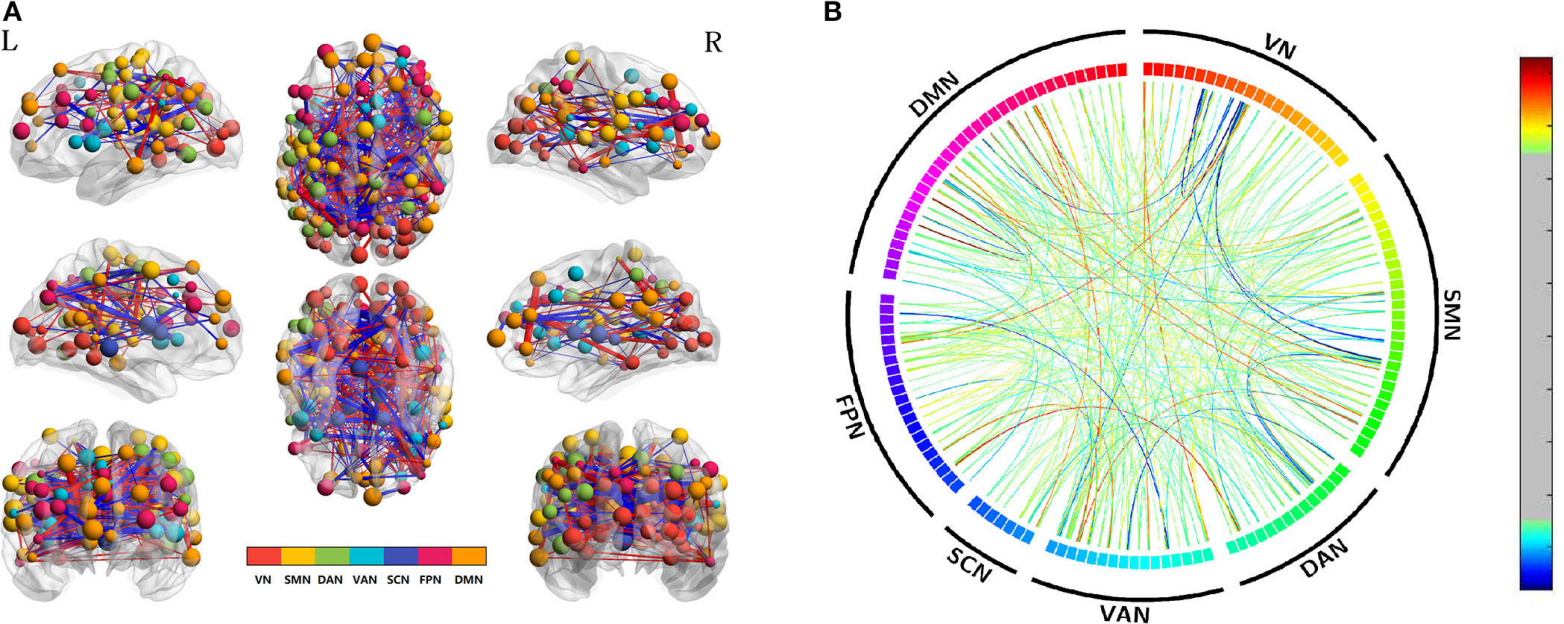
Altered edge-based functional connectivity for acute SD subjects between after and before acupuncture. **(A)** The brain maps show the affected edges (lines) and their connecting nodes (spheres) from several perspectives. The size of a node indicates how many affected edges are connected to this sphere. Bigger nodes have more affected edges than smaller ones. The color of a node indicates which network it belongs to. The warm color of edges indicates the increased FC after acupuncture, while the cold color of edges indicates the decreased FC. **(B)** ROI to ROI connectivity of seven networks in panels. The result was corrected by NBS (*p* < 0.01, two-tailed, permutation with 5,000 iterations). DAN, dorsal attention network; DMN, default mode network; FC, functional connectivity; FPN, frontoparietal network; L, left; R, right; SCN, subcortical network; SMN, somatosensory network; VAN, ventral attention network; VN, visual network; SD, sleep deprivation.

To explore acupuncture effects on acute SD, we then examined the differences in edge-based FC for SD between before and after acupuncture at HT7. Compared with SD before acupuncture at HT7, we found the suprathreshold edges with abnormal within-network FC involving all brain networks except SCN, and both decreased and increased between-network FC of edges involving all paired networks except FPN-SCN. Moreover, we found that SD subjects demonstrated only increased within-network FC of the DAN after acupuncture at HT7, while both increased and decreased within-network FC of the VN, SMN, VAN, FPN, and DMN. The details were shown in Table 2 and Figure 3.

**Table 2:** Number and ratio of decreased and increased ROI-wise FC for acute SD subjects between after and before acupuncture.

**Number**	**VN**	**SMN**	**DAN**	**VAN**	**SCN**	**FPN**	**DMN**
**Percent**							
**Decreased ROI-wise FC**							
VN	3						
	1.30%						
SMN	11	8					
	1.72%	1.97%					
DAN	7	10	2				
	2.27%	2.46%	2.20%				
VAN	12	6	7	3			
	3.41%	1.29%	3.13%	2.50%			
SCN	1	3	4	3	0		
	0.65%	1.48%	4.08%	2.68%	0.00%		
FPN	3	5	3	8	0	4	
	0.65%	0.82%	1.02%	2.38%	0.00%	1.90%	
DMN	6	16	5	14	4	9	11
	0.83%	1.67%	1.08%	2.65%	1.73%	1.30%	2.08%
**Increased ROI-wise FC**							
VN	2						
	0.87%						
SMN	6	5					
	0.94%	1.23%					
DAN	7	4	0				
	2.27%	0.99%	0.00%				
VAN	4	11	3	3			
	1.14%	2.37%	1.34%	2.50%			
SCN	2	3	2	1	0		
	1.30%	1.48%	2.04%	0.89%	0.00%		
FPN	12	11	7	7	4	2	
	2.60%	1.81%	2.38%	2.08%	2.72%	0.95%	
DMN	9	18	7	12	1	13	5
	1.24%	1.88%	1.52%	2.27%	0.43%	1.88%	0.95%

*Values in the first line of each cell are the count number of suprathreshold edges belonging to each pair of networks for the significant cluster obtained from the NBS analysis; while values in the second line are the ratio (in percent) of that number to the number of full connections for each pair of networks. VN, visual network; SMN, somatosensory network; DAN, dorsal attention network; VAN, ventral attention network; SCN, subcortical network; FPN, frontoparietal network; DMN, default mode network; FC, functional connectivity; RW,SD, sleep deprivation; ROI, region of interest*.

**Figure 4:**
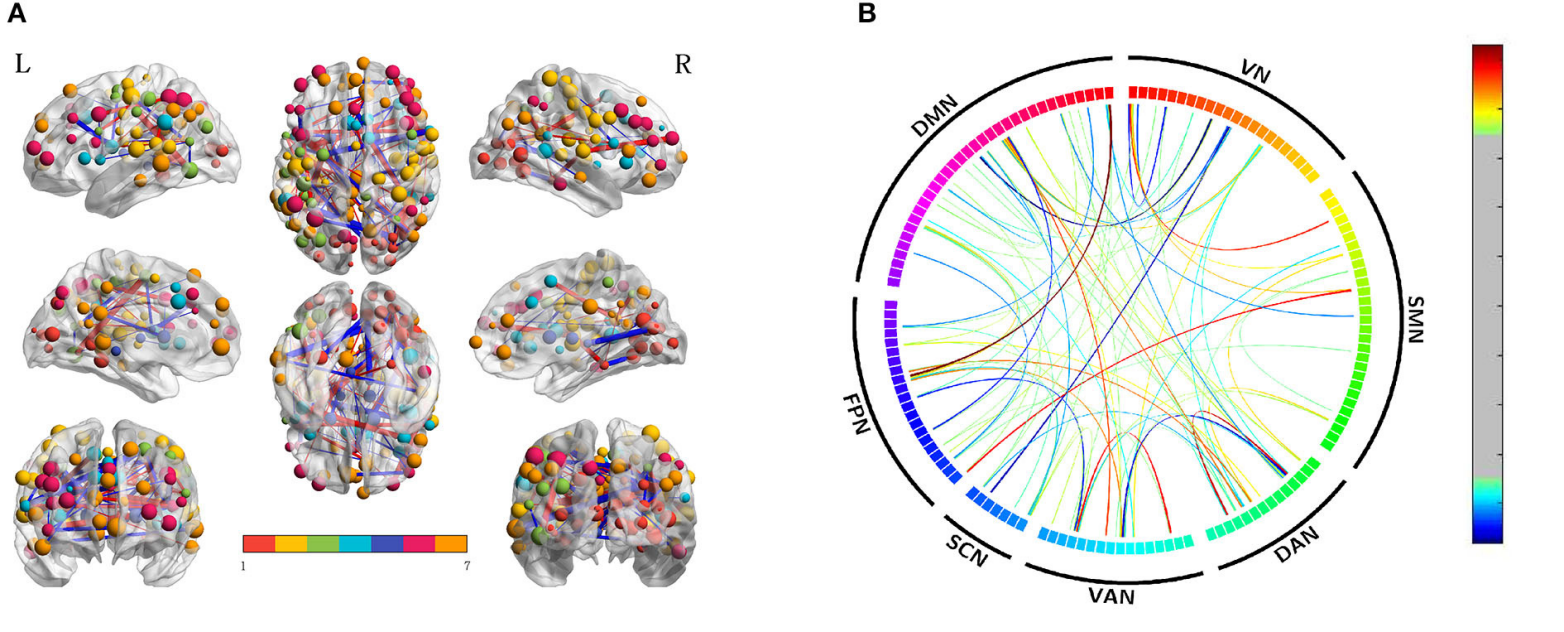
Altered edge-based functional connectivity between the acute SD after acupuncture and RW state. **(A)** The brain maps show the affected edges (lines) and their connecting nodes (spheres) from several perspectives. The size of a node indicates how many affected edges are connected to this sphere. Bigger nodes have more affected edges than smaller ones. The color of a node indicates which network it belongs to. The warm color of edges indicates the increased FC after acupuncture, while the cold color of edges indicates the decreased FC. **(B)** ROI to ROI connectivity of seven networks in panels. The result was corrected by NBS (*p* < 0.01, two-tailed, permutation with 5,000 iterations). DAN, dorsal attention network; DMN, default mode network; FC, functional connectivity; FPN, frontoparietal network; L, left; R, right; SCN, subcortical network; SMN, somatosensory network; VAN, ventral attention network; VN, visual network; RW, rested wakefulness.

We also compared the differences between SD after acupuncture and RW state. Compared with SD, we found both decreased and increased between-network FC of suprathreshold edges involving DAN only and decreased or increased between-network FC of edges involving all paired networks except SMN-SCN. The details are shown in Table 3 and Figure 4.

**Table 3:** Number and ratio of decreased and increased ROI-wise FC between acute SD after acupuncture and RW state.

**Number**	**VN**	**SMN**	**DAN**	**VAN**	**SCN**	**FPN**	**DMN**
**Percent**							
**Decreased ROI-wise FC**							
VN	0						
	0.00%						
SMN	3	0					
	0.47%	0.00%					
DAN	1	3	1				
	0.32%	0.74%	1.10%				
VAN	1	2	0	1			
	0.28%	0.43%	0.00%	0.83%			
SCN	0	0	0	2	0		
	0.00%	0.00%	0.00%	1.79%	0.00%		
FPN	1	1	2	2	0	0	
	0.22%	0.16%	0.68%	0.60%	0.00%	0.00%	
DMN	3	0	7	3	1	5	0
	0.41%	0.00%	1.52%	0.57%	0.43%	0.72%	0.00%
**Increased ROI-wise FC**							
VN	2						
	0.87%						
SMN	0	1					
	0.00%	0.25%					
DAN	2	0	1				
	0.65%	0.00%	1.10%				
VAN	1	1	3	0			
	0.28%	0.22%	1.34%	0.00%			
SCN	1	0	1	0	0		
	0.65%	0.00%	1.02%	0.00%	0.00%		
FPN	2	1	0	2	2	2	
	0.43%	0.16%	0.00%	0.60%	1.36%	0.95%	
DMN	5	1	3	1	4	4	3
	0.69%	0.10%	0.65%	0.19%	1.73%	0.58%	0.57%

*Values in the first line of each cell are the count number of suprathreshold edges belonging to each pair of networks for the significant cluster obtained from the NBS analysis; while values in the second line are the ratio (in percent) of that number to the number of full connections for each pair of networks. VN, visual network; SMN, somatosensory network; DAN, dorsal attention network; VAN, ventral attention network; SCN, subcortical network; FPN, frontoparietal network; DMN, default mode network; FC, functional connectivity; RW,SD, sleep deprivation; ROI, region of interest*.

### Large-scale network FC

**Figure 5:**
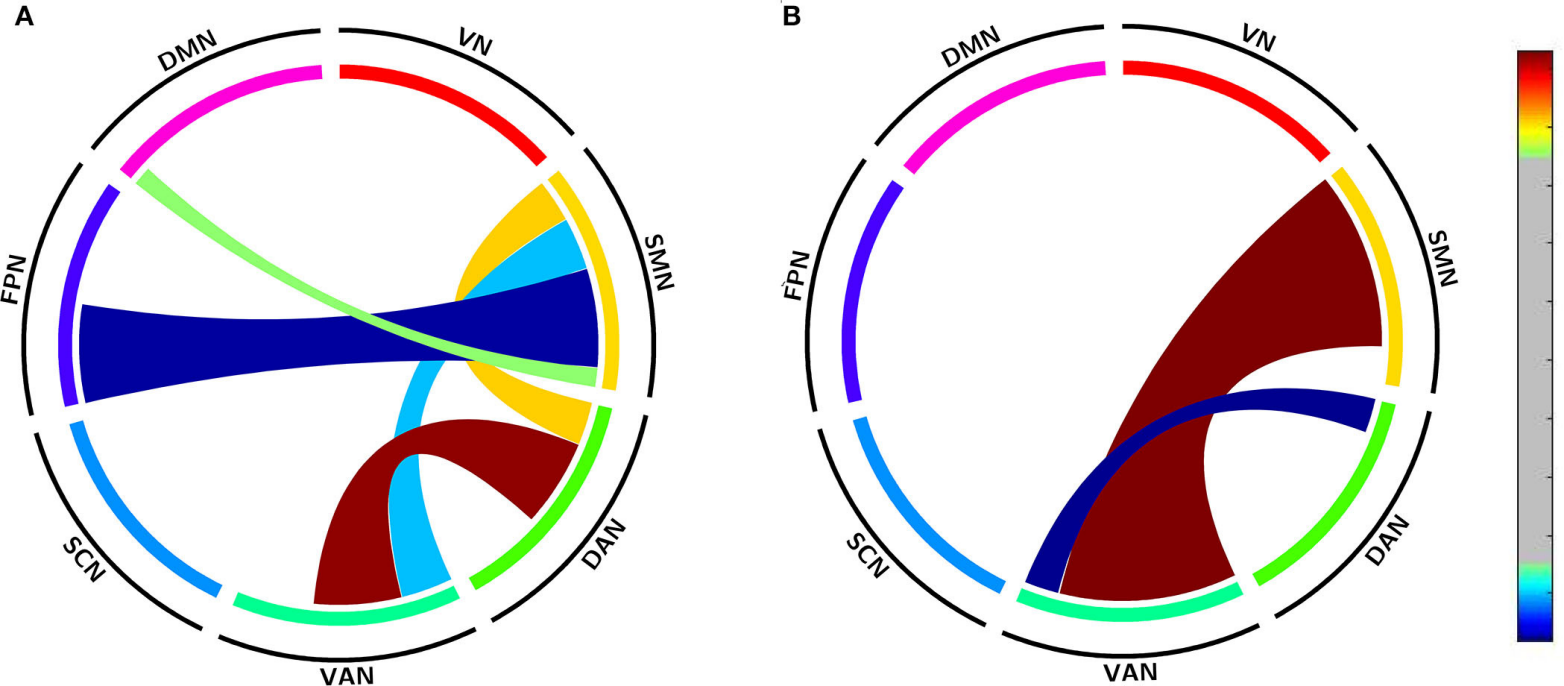
Altered large-scale network functional connectivity. **(A)** Altered large-scale network functional connectivity between SD and RW state; **(B)** Altered large-scale network functional connectivity for SD between after and before acupuncture. For the *T*-value color bar, blue indicates functional connectivity decrease while red indicates functional connectivity increase. The result was corrected by FDR-corrected *p* < 0.05 (two-tailed). DAN, dorsal attention network; DMN, default mode network; FC, functional connectivity; FPN, frontoparietal network; L, left; R, right; SCN, subcortical network; SMN, somatosensory network; VAN, ventral attention network; VN, visual network; RW, rested wakefulness; SD, sleep deprivation.

To validate acupuncture effects, we furtherly analyzed large-scale within- and between-network FC. First, we compared the differences in large-scale network FC between the acute SD and RW states. We found that SD subjects showed a significant decrease in network FC in SMN-DMN, SMN-FPN, and SMN-VAN, and increased between-network FC in DAN-VAN, DAN-SMN. No abnormal within-network FC was found in acute SD subjects. We then conducted the comparisons between before and after acupuncture at HT7. Results revealed that the SD subjects showed significantly decreased between-network FC in DAN-VAN, increased between-network FC in SMN-VAN after acupuncture at HT7. There was no altered within-network FC in SD subjects after acupuncture. At last, we compared large-scale within- and between-network FC between acute SD after acupuncture and RW, and found no significant difference between the two states. The details are shown in Table 4 and Figure 5.

**Table 4:** Large scale between-network FC changes.

**Between networks**	***t***	***p***
**SD** **>** **RW**		
VAN-SMN	−2.18	0.04
SMN-DAN	2.15	0.04
SMN-VAN	−2.18	0.04
DAN-VAN	2.32	0.03
SMN-FPN	−2.36	0.03
SMN-DMN	−2.05	0.05
**After > before acupuncture**		
VAN-SMN	2.8	0.01
VAN-DAN	−2.3	0.03

*VN, visual network; SMN, somatosensory network; DAN, dorsal attention network; VAN, ventral attention network; SCN, subcortical network; FPN, frontoparietal network; DMN, default mode network, FC, functional connectivity; RW, rested wakefulness; SD, sleep deprivation*.

## Discussion

In the current study, we recruited 30 healthy subjects with regular sleep to compare their network FC between RW state and after 24 h of total SD, and before and after acupuncture at HT7. The edge-based results showed the suprathreshold edges with abnormal between-network FC involving all paired networks except SMN-SCN between the RW and after 24 h of total SD, while both decreased and increased between-network FC of edges involving all paired networks except FPN-SCN between before and after acupuncture at HT7. Compared with the RW state, the large-scale brain network results showed decreased between-network FC in SMN-DMN, SMN-FPN, SMN-VAN, and increased between-network FC in DAN -VAN, DAN-SMN between the RW state and after 24 h of total SD. After acupuncture at HT7, the large-scale brain network results showed decreased between-network FC in DAN-VAN, and increased between-network FC in SMN-VAN. In a word, our results may preliminarily provide new evidence to interpret the effect of acupuncture at HT7 on acute SD individuals.

In comparison with RW subjects, the FC in widespread brain regions and brain networks altered after 24 h of SD in our study. In line with our results, previous studies had demonstrated that functional brain networks in SD were abnormal involving SMN, DMN, Salience Network (SN), DAN, and FPN (17, 18, 31). A graph theory-based study on the whole brain networks also revealed that the small-world property of resting-state networks was significantly enhanced after acute SD (32). DMN is an internally directed network, which has been reported decreased FC within the network after acute SD (17). It is important that DMN can impact rest-stimulus interactions in corresponding sensory cortices (33), which may explain the decreased FC in DMN-SMN after SD. Also, it had been observed that SD could impact DAN which is associated with the top-down deployment of attention (34, 35). Several fMRI studies have revealed that SD could change the intrinsic connectivity within the DAN and its related anti-correlated network (i.e., DMN) (17, 20). FPN, which mainly supports the control of information processing, can contribute to verbal expression, memory, and cognitive control (36). After acute SD, the decreased FC in FPN-DMN was found, which was associated with working-memory performance (18). Notably, the SMN was found to be more affected (more pairs of large-scale networks) than other networks in the current study. Brain regions of the SMN control motor, somatosensory and auditory processing, and are responsible for external stimuli and internally generated movement. It has been demonstrated that the brain networks related to sleep and wake are modulated by sensory inputs, meanwhile, both sensory information and deprivation may induce changes in brain networks relative to sleep and waking up (37). One study revealed the decreased FC between the putamen and the main brain regions of SMN after acute SD, which in turn impaired motor perception and fine motor control. Another previous study also illustrated that the connection between the SMN and SN was reduced after acute SD (38). Thus, our results may support the notion that the SMN is the core network of altered large scale networks due to sleep loss. In short, we speculated that the abnormal FC within and between networks after acute SD might explain SD-induced impairments in cognitions and emotional discrimination, and be interpreted as a possible compensatory adaptation of the human brain that could enable partial recovery of certain behaviors.

Acupuncture, as the key component of Traditional Chinese Medicine, has been reported to modulate FC across the brain networks (8, 39, 40). Previous neuroimaging studies have also illustrated remarkable changes in brain activities responsive to acupuncture at the “Shenmen” point intervening on SD (10, 41). In this study, we found all seven networks were affected by acupuncture at HT7. According to previous neuroimaging studies, acupuncture stimuli could modulate extensive brain regions involving somatosensory, cognitive, and affective processing (8). A meta-analysis study on brain activities responding to acupuncture revealed that acupuncture could activate SMN and deactivate the limbic-paralimbic neocortical network (42). Apart from the activation/deactivation, the FC within and between networks could also be modulated by acupuncture (43, 44). These findings demonstrated that acupuncture could modulate multi-scale brain functions across the brain regions and networks, which was in line with our edge-based results. Moreover, we found the decreased FC in DAN-VAN and increased FC in SMN-VAN by large-scale network analysis. The DAN controls goal-oriented top-down deployment of attention (45), while the VAN partly overlapping with SN mediates stimulus-driven bottom-up attentional reorienting (46). The interaction between the two networks was competitive among multiple stimuli in the visual cortex and mediated the selection of behaviorally relevant information (47). One study on major depressive disorders revealed the increased clustering coefficient and small-worldness of DAN and VAN (48). Another study on healthy subjects demonstrated granger causal influences from VAN to DAN are negatively associated with attention performance (49). Hence, we speculated that the decreased FC between VAN and DAN might be interpreted as the treatment effect on the attention deficits of SD subjects. Regions of the SMN are spatially adjacent to regions of the DAN and VAN in the brain and cooperate with DAN and VAN during external tasks (30). The decreased FC in SMN-VAN after 24 h of total SD suggested abnormal performances during cognitive tasks. Previous neuroimaging studies confirmed that acupuncture could modulate SMN in low back pain and stroke patients, which represented the effects of acupuncture on diseases (50, 51). Thus, the enhanced FC in SMN-VAN after acupuncture could be interpreted as the treatment effects on acute SD. Together with changes in the DAN-VAN and SMN-VAN, no change between DMN and other networks (no significant pairs of large-scale networks) may suggest that acupuncture mainly modulates intrinsic FC of the externally directed networks. Taken together, the altered FC in SD-induced brain networks after acupuncture may indicate the mechanism of acupuncture on acute SD.

As to the analysis method in the current study, the surface-based preprocessing method was applied, which was better than the volume-based method for registration, reproducibility of algorithms, and surface reconstructions (52). Differing from previous neuroimaging studies on SD by employing seed-based FC analysis (18, 53), we applied both NBS and large-scale brain network analyses to explore SD-related abnormalities and acupuncture effects on intrinsic FC in large-scale brain networks. Hereby, our results could reveal the altered FC of large-scale brain networks after acupuncture at HT7.

However, there were still some limitations. First, as a preliminary study to explore the effect of acupuncture on SD, we only investigated the immediate effect at a single acupoint. Further studies on the long-term effects of acupuncture with group acupoints will be needed to explore the relationship between cognitive function improvements and FC changes. Secondly, without a control group of SD subjects with sham acupuncture treatment, we could not precisely quantify the effect of acupuncture on large-scale brain network FC. Nevertheless, in line with previous neuroimaging studies without the control group (22, 25, 54), our results indicated the possible mechanisms of the modulatory effects of acupuncture on SD. Furthermore, it is a challenge to set up sham acupuncture, which is not only difficult to implement but also has some specific efficacy, thus underestimating the therapeutic effect of acupuncture in the trial (55). Finally, numerous studies suggested that longer resting-state scans could improve reliability and replicability (56, 57). In the current study, we collected 490-s resting-state data. Future studies with longer resting-state scans will be needed to validate our results.

## Conclusion

The edge-based results detected both decreased and increased between-network FC of edges involving most of the paired networks after acupuncture at HT7. The large-scale brain network results demonstrated that acupuncture could reverse the altered between-network FC of DAN-VAN and SMN-VAN. In short, acupuncture could widely modulate all brain networks and reverse the specific between-network FC.

## Data availability statement

The raw data supporting the conclusions of this article will be made available by the authors, without undue reservation.

## Ethics statement

The studies involving human participants were reviewed and approved by Ethics Committee of Beijing Anding Hospital. The patients/participants provided their written informed consent to participate in this study.

## Author contributions

YN and HJ: conception and design. SZ, HY, ZF, XL, and LD: data collection. YN and SZ: data analysis. YN and SF: writing. SF: english-language revision. HJ and YN: revision. All authors contributed to the final version of the manuscript and approved the submitted version.

## Funding

This study was supported by National Natural Science Foundation (Grant no. 81904120), Beijing Hospitals Authority Youth Program (Grant no. QML20201901), Beijing Natural Science Foundation (Grant no. 7212050), Beijing Hospitals Authority Clinical Medicine Development of Special Funding (Grant no. ZYLX202129), Beijing Hospitals Authority's Ascent Plan (Grant no. DFL20191901), and Talents Training Fund of Beijing (Grant no. 2018000021469G292).

## Conflict of interest

The authors declare that the research was conducted in the absence of any commercial or financial relationships that could be construed as a potential conflict of interest.

## Publisher's note

All claims expressed in this article are solely those of the authors and do not necessarily represent those of their affiliated organizations, or those of the publisher, the editors and the reviewers. Any product that may be evaluated in this article, or claim that may be made by its manufacturer, is not guaranteed or endorsed by the publisher.
